# Not all that glitters is COVID! Differential diagnosis of FDG-avid interstitial lung disease in low-prevalence regions

**DOI:** 10.1186/s41824-020-00088-6

**Published:** 2020-10-19

**Authors:** Annalisa Papa, Chiara Pozzessere, Francesco Cicone, Fabiola Rizzuto, Giuseppe Lucio Cascini

**Affiliations:** 1 Nuclear Medicine Unit, University Hospital “Mater Domini”, Catanzaro, Italy; 2 Radiology Unit, AUSL Toscana Centro San Giuseppe Hospital, Empoli, Italy; 3 Department of Experimental and Clinical Medicine, “Magna Graecia” University of Catanzaro, Viale Europa – 88100, Catanzaro, Italy; 4 Medical Oncology Unit, Hospital “Pugliese Ciaccio”, Catanzaro, Italy

**Keywords:** COVID-19, FDG PET/CT, SARS-CoV-2, Differential diagnosis, Pneumonia, Radiological features

## Abstract

Coronavirus disease-19 (COVID-19) is only one of the many possible infectious and non-infectious diseases that may occur with similar imaging features in patients undergoing [^18^F]-fluorodeoxyglucose (^18^FDG) monitoring, particularly in the most fragile oncologic patients. We briefly summarise some key radiological elements of differential diagnosis of interstitial lung diseases which, in our opinion, could be extremely useful for physicians reporting ^18^FDG PET/CT scans, not only during the COVID-19 pandemic, but also for their normal routine activity.

During the recent pandemic, several papers have reported on the [^18^F]-fluorodeoxyglucose positron-emission tomography (^18^FDG PET) findings in coronavirus disease-19 (COVID-19). Increased FDG uptake is usually seen at the site of lung abnormalities shown on computed tomography (CT) and may also be present in mediastinal lymph nodes (O’Neill et al. 2020; Polverari et al. 2020). This is not unexpected since ^18^FDG avidity is typical of inflammatory/infectious diseases, and some authors have proposed ^18^FDG PET as a non-invasive biomarker for monitoring viral infections (Brust et al. 2006; Chacko et al. 2017). A paper suggested that ^18^FDG uptake by lymph nodes might have a prognostic role in patients with COVID-19 (O’Neill et al. 2020). However, until now, ^18^FDG PET/CT is not recommended in the workup of suspected or confirmed COVID-19, and the reported cases of ^18^FDG PET/CT in patients with COVID-19 are most often incidental (Treglia 2020). The severe acute respiratory syndrome coronavirus 2 (SARS-CoV-2) spread has been remarkably heterogeneous so far, which has relevant implications for the interpretation of indeterminate imaging findings. In regions where prevalence is high, it is reasonable to first suspect the diagnosis of COVID-19 for otherwise healthy individuals presenting with upper respiratory symptoms and/or characteristic imaging findings (Pozzessere et al. 2020a). Conversely, different considerations should be made in regions where COVID-19 has a lower prevalence, particularly for patients undergoing ^18^FDG PET monitoring during or shortly after anticancer treatments. We recently observed ^18^FDG-avid lung abnormalities suspected for COVID-19 in a symptomatic male patient with advanced squamous cell lung carcinoma, who was referred for ^18^FDG PET/CT at the end of first-line chemotherapy with cisplatin-gemcitabine combination (Fig. 1). Of note, our institution is located in Calabria, a southern Italian region where the diffusion of SARS-CoV-2 was very limited compared to northern Italian regions. As of July 14, 2020, official data report a COVID-19 cumulative incidence of 61.68 vs. 945.45 per 100,000 people for Calabria and Lombardy, the most affected Italian region, respectively (Istituto superiore di sanità pubblica. Epidemia COVID-19. Aggiornamento nazionale 14 Luglio 2020). In this patient, two nasopharyngeal swabs tested negative for SARS-CoV-2. Finally, serum negativity for anti-SARS-CoV-2 IgM and IgG antibodies confirmed that the patient had never been exposed to the infection. Regretfully, the patient’s conditions worsened rapidly and death occurred before a definitive etiological diagnosis could be made. This case reminded us that, while a prompt recognition of COVID-19 is critically important in order to limit the diffusion of the disease among the healthcare staff and the general population, physicians reporting hybrid PET/CT scans should also keep alert for all serious conditions that may present with similar imaging features, particularly in the most fragile patients.

**Fig. 1:**
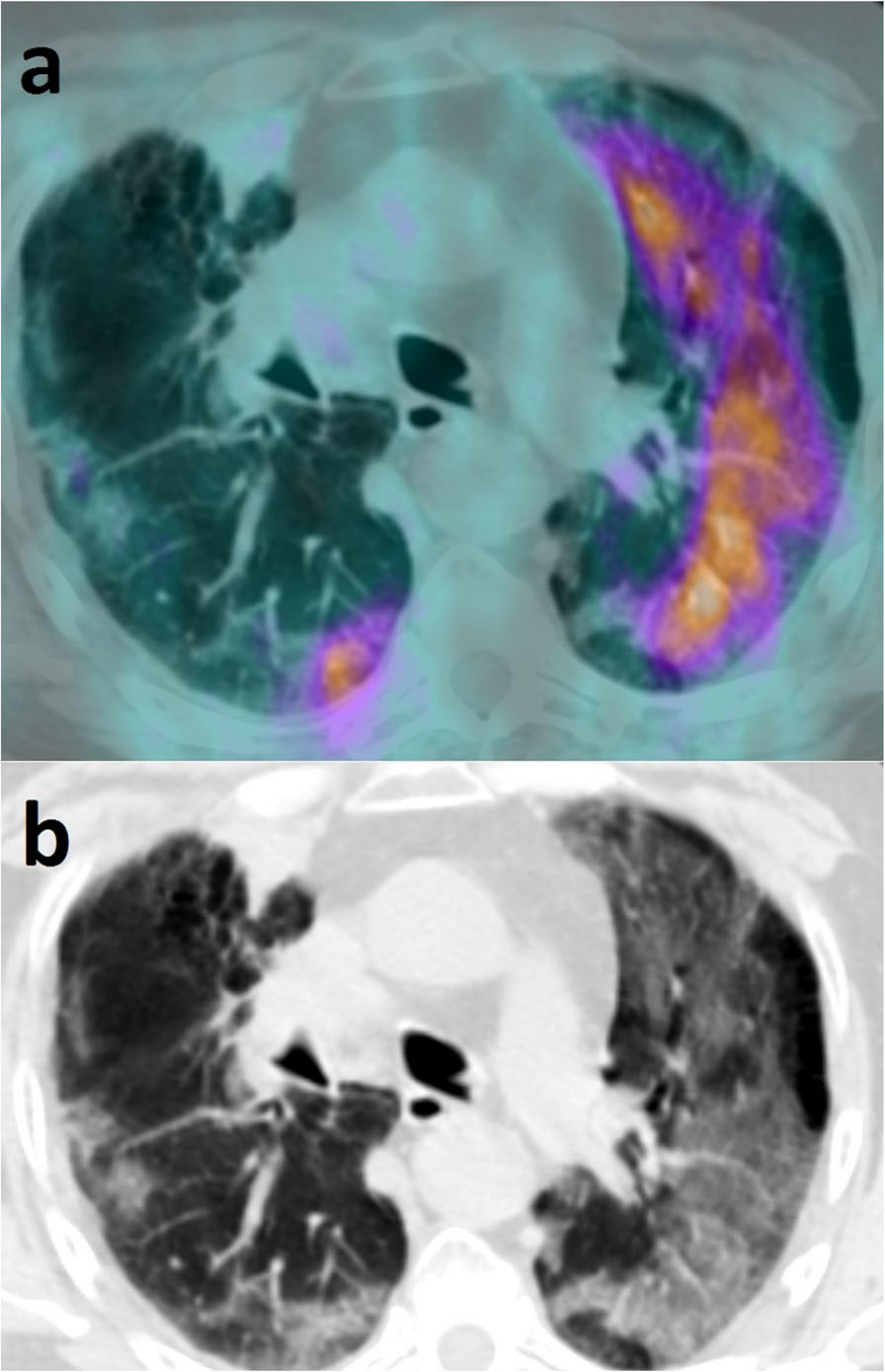
**a** Axial PET/CT view of the thorax showing diffuse, non-homogeneously increased FDG uptake (maximum SUV = 5) at the site of bilateral lung abnormalities. Additional images of pathologically increased FDG uptake were seen at the site of the primary tumor, in the apex of the right lung, and at the sites of multiple bone and liver metastases (not shown). No FDG-avid lymph-nodes were detected. **b** Corresponding CT view showing multifocal patchy centro-parenchymal and subpleural GGOs with subpleural fibrotic stripes of the right lung, as well as diffuse GGOs of the left lung with partial sparing of the subpleural space. Bronchial wall thickening and some bronchiectasis were also seen. No consolidations or micronodules, nor cysts or pleural effusion were detected. These features, in addition to the asymmetrical appearance and the sparing of the apical segments, orient, in first instance, towards an interstitial pneumonia of viral aetiology. In second instance, a pneumonitis induced by cisplatin-gemcitabine should be considered, as this is also characterized by multifocal or diffuse GGOs. Furthermore, although imaging features are not highly suspicious for *Pneumocystis Jirovecii*, the latter still represents a possible alternative diagnosis

The main radiological features of COVID-19 pneumonia include multifocal or diffuse bilateral ground-glass opacities (GGOs) and “crazy-paving” pattern with a predominant subpleural distribution (Hani et al. 2020; Salehi et al. 2020). At the early stage, patchy centro-parenchymal GGOs have been described (Pozzessere et al. 2020a). As the disease progresses, GGOs coalesce into consolidations which may cavitate in the most severe cases (Hani et al. 2020; Salehi et al. 2020). The recovery phase is characterized by the reduction in size and density of both consolidations and GGOs, with the appearance of parenchymal stripes (Salehi et al. 2020). However, these findings are not pathognomonic of COVID-19, and several differential diagnoses should be considered, especially in vulnerable patients such as oncologic patients. Similar imaging features can be found in other infectious and non-infectious diseases, all of which can show variable degrees of ^18^FDG uptake. These include common viral infections of the lower respiratory tract, such as influenza virus, especially H1N1, parainfluenza virus, adenovirus, respiratory syncytial virus (RSV), and opportunistic infections, arising in severely immunocompromised patients, such as *Pneumocystis jirovecii*, cytomegalovirus (CMV), herpes simplex virus (HSV), varicella-zoster virus (VZV), or Mycoplasma pneumoniae (Beigelman-Aubry et al. 2012). Although imaging findings substantially overlap between COVID-19 and these diseases, some subtle radiological features might help orient the diagnosis.

During the flu season, H1N1 pneumonia represents the main differential diagnosis, particularly in case of bilateral consolidations and GGOs with a predominant peripheral and peribronchovascular distribution. However, GGOs are more represented in COVID-19 than in H1N1 pneumonia (Tang et al. 2020). Possible additional differences are the presence of pleural effusion, bronchiectasis, and bronchial wall-thickening, which are more frequent in patients with H1N1, whereas “crazy-paving” pattern is more common in patients with COVID-19 (Yin et al. 2020). When bronchial wall thickening and micronodules in a “tree in bud” distribution are found in association with limited GGOs and consolidations, other lower respiratory tract infections such as RSV, adenovirus, and parainfluenza should be considered (Miller Jr et al. 2011). When bilateral, diffuse GGOs or “crazy-paving” pattern is detected in immunocompromised patients, opportunistic infections are the most fearsome differential diagnoses. Severe COVID-19 lung involvement and *Pneumocystis jirovecii* pneumonia (PCP) may show identical imaging features, that is bilateral and diffuse GGOs and/or “crazy-paving” pattern. However, different from COVID-19, the subpleural space is spared in half of the patients with PCP, and thin-wall cysts may be occasionally seen (Kanne et al. 2012). Moreover, PCP has a predominant apical distribution, while COVID-19 pneumonia involves more extensively the basal regions. In CMV, HSV, and HZV, diffuse bilateral GGOs or “crazy-paving” pattern may be associated with centrilobular nodules, which have not been described in COVID-19 (Beigelman-Aubry et al. 2012). Furthermore, patients receiving anti-cancer treatments may develop drug-induced pneumonia with interstitial patterns including GGOs and reticulations that, if untreated, may lead to pulmonary fibrosis (Torrisi et al. 2011). Several cytotoxic and targeted therapies may cause lung injury; among these, there are bleomycin, methotrexate, gemcitabine, paclitaxel, oxaliplatin, mammalian target of rapamycin (mTOR) inhibitors, anti-epidermal growth factor receptor (EGFR) antibodies, and immune check-point inhibitors (ICIs) (Torrisi et al. 2011; Naidoo et al. 2017). In particular, immune-related adverse events induced by ICIs may show imaging findings similar to COVID-19 pneumonia, especially when the lung involvement is diffuse and complicated by diffuse alveolar damage/acute respiratory distress syndrome (DAD/ARDS) pattern (Naidoo et al. 2020; Pozzessere et al. 2020b). The diagnosis of drug-induced toxicity is often suspected after infectious pneumonia, and other lung diseases have been excluded. It should be acknowledged that integrated PET/CT systems have known limitations in the study of the lung parenchyma, including free-breathing acquisitions and suboptimal spatial resolution, which make a correct differential diagnosis difficult in clinical practice. Notwithstanding this, in our opinion, the key radiological features we have briefly summarised should be part of the cultural experience of the physician reporting hybrid PET/CT scans, in order to advise the referring clinician on the correct patient management.

## Data Availability

Not applicable

## Abbreviations

^18^FDG: [^18^F]-Fluorodeoxyglucose
PET: Positron-emission tomography
COVID-19: Coronavirus disease-19
CT: Computed tomography
SARS-CoV-2: Severe acute respiratory syndrome coronavirus 2
GGOs: Ground-glass opacities
RSV: Respiratory syncytial virus
CMV: Cytomegalovirus
HSV: Herpes simplex virus
VZV: Varicella-zoster virus
PCP: *Pneumocystis jirovecii* pneumonia
mTOR: Mammalian target of rapamycin
EGFR: Epidermal growth factor receptor
ICIs: Immune check-point inhibitors
DAD/ARDS: Diffuse alveolar damage/acute respiratory distress syndrome
